# *Urtica dioica*-Derived Phytochemicals for Pharmacological and Therapeutic Applications

**DOI:** 10.1155/2022/4024331

**Published:** 2022-02-24

**Authors:** Yasaman Taheri, Cristina Quispe, Jesús Herrera-Bravo, Javad Sharifi-Rad, Shahira M. Ezzat, Rana M. Merghany, Shabnum Shaheen, Lubna Azmi, Abhay Prakash Mishra, Bilge Sener, Mehtap Kılıç, Surjit Sen, Krishnendu Acharya, Azadeh Nasiri, Natália Cruz-Martins, Patrick Valere Tsouh Fokou, Alibek Ydyrys, Zhandos Bassygarayev, Sevgi Durna Daştan, Mohammed M. Alshehri, Daniela Calina, William C. Cho

**Affiliations:** ^1^Phytochemistry Research Center, Shahid Beheshti University of Medical Sciences, Tehran, Iran; ^2^Facultad de Ciencias de la Salud, Universidad Arturo Prat, Avda. Arturo Prat 2120, Iquique 1110939, Chile; ^3^Departamento de Ciencias Básicas, Facultad de Ciencias, Universidad Santo Tomas, Chile; ^4^Center of Molecular Biology and Pharmacogenetics, Scientific and Technological Bioresource Nucleus, Universidad de La Frontera, Temuco, 4811230, Chile; ^5^Facultad de Medicina, Universidad del Azuay, Cuenca, Ecuador; ^6^Department of Pharmacognosy, Faculty of Pharmacy, Cairo University, Kasr El Ainy Street, Cairo 11562, Egypt; ^7^Department of Pharmacognosy, Faculty of Pharmacy, October University for Modern Sciences and Arts (MSA), 6th of October 12451, Egypt; ^8^Department of Pharmacognosy, National Research Centre, Giza, Egypt; ^9^Department of Plant Sciences, LCWU, Lahore 54000, Pakistan; ^10^Hygia Institute of Pharmaceutical Education & Research, Lucknow, U. P. 226001, India; ^11^Department of Pharmacology, University of Free State, Bloemfontein 9300, Free State, South Africa; ^12^Gazi University, Faculty of Pharmacy, Department of Pharmacognosy, Ankara 06330, Turkey; ^13^Department of Pharmacognosy, Lokman Hekim University Faculty of Pharmacy, Ankara 06510, Turkey; ^14^Molecular and Applied Mycology and Plant Pathology Laboratory, Department of Botany, University of Calcutta, Kolkata 700019, India; ^15^Department of Botany, Fakir Chand College, Diamond Harbour, West Bengal 743331, India; ^16^Department of Pharmacology and Toxicology, School of Pharmacy, Shahid Beheshti University of Medical Sciences, Tehran, Iran; ^17^Faculty of Medicine, University of Porto, Porto, Portugal; ^18^Institute for Research and Innovation in Health (i3S), University of Porto, Porto, Portugal; ^19^Institute of Research and Advanced Training in Health Sciences and Technologies (CESPU), Rua Central de Gandra, 1317, Gandra PRD 4585-116, Portugal; ^20^TOXRUN-oxicology Research Unit, University Institute of Health Sciences, CESPU, CRL, Gandra 4585-116, Portugal; ^21^Department of Biochemistry, Faculty of Science, University of Bamenda, Bambili, P.O. Box. 39, Cameroon; ^22^Biomedical Research Centre, Al-Farabi Kazakh National University, Al-Farabi av. 71, Almaty 050040, Kazakhstan; ^23^Department of Biophysics, Biomedicine and Neuroscience, Al-Farabi Kazakh National University, Al-Farabi av. 71, Almaty 050040, Kazakhstan; ^24^Department of Biology, Faculty of Science, Sivas Cumhuriyet University, Sivas 58140, Turkey; ^25^Beekeeping Development Application and Research Center, Sivas Cumhuriyet University, Sivas 58140, Turkey; ^26^Pharmaceutical Care Department, Ministry of National Guard-Health Affairs, Riyadh, Saudi Arabia; ^27^Department of Clinical Pharmacy, University of Medicine and Pharmacy of Craiova, Craiova 200349, Romania; ^28^Department of Clinical Oncology, Queen Elizabeth Hospital, Kowloon, Hong Kong

## Abstract

*Urtica dioica* belongs to the Urticaceae family and is found in many countries around the world. This plant contains a broad range of phytochemicals, such as phenolic compounds, sterols, fatty acids, alkaloids, terpenoids, flavonoids, and lignans, that have been widely reported for their excellent pharmacological activities, including antiviral, antimicrobial, antihelmintic, anticancer, nephroprotective, hepatoprotective, cardioprotective, antiarthritis, antidiabetic, antiendometriosis, antioxidant, anti-inflammatory, and antiaging effects. In this regard, this review highlights fresh insight into the medicinal use, chemical composition, pharmacological properties, and safety profile of *U. dioica* to guide future works to thoroughly estimate their clinical value.

## 1. Introduction

Genus *Urtica,* commonly known as “nettle,” is a medicinal plant belonging to the family Urticaceae with multiple health benefits that have been used medicinally since at least the times of Ancient Greece [1]. Several *Urtica* species have been widely used to treat rheumatism and sciatica, asthma, coughs, dandruff, diabetes, diarrhea, eczema, fever, gout, hemorrhoids, nose bleeds, scurvy, snake bites, and tuberculosis [2]. Moreover, *Urtica* species have been used most commonly as a diuretic and for treating gout, anemia, and prostate hypertrophy, with several studies progressively reporting their traditional medicinal use by local people [2–11].

These studies originated mostly from African, European, Asian, and Oceanian countries, such as Algeria, Argentina, Australia, Bolivia, Bhutan, Brazil, Bolivia, Belarus, Bolivia, Canada, Chile, China, Colombia, Cyprus, Costa Rica, Cuba, Ecuador, Egypt, France, Guatemala, India, Italy, Israel, Japan, Korea, Mexico, Nepal, New Zealand, Netherlands, North America, Palestine, Paraguay, Peru, Russia, Sikkim, Sweden, Spain, Taiwan, Turkey, Tunisia, United States, Uruguay, Ukraine, and Vietnam [2–11].

Data obtained from these studies mostly underlined that *Urtica* species exert excellent antirheumatoid arthritis, antigout, anti-inflammatory, immunomodulatory, and antioxidant activities, all of which contribute to the protection of joints. In addition, it has been revealed to be extremely useful for the treatment of microbial and parasitic infections, cancer, jaundice, stomach diseases, snakebites, diabetes, liver and kidney problems, wounds, diuretic, libido, pulmonary diseases, hypotensive, blood purification, urticaria, allergic rhinitis, prostate disorders, hemorrhoids, and galactagogue and as a depurative. Apart from this, these species have also been reported to be used for exorcism, postcalving care, sprains, bones fracture, hematuria, neck sore, and yolk sore [2, 12–26].

Despite the scientific advances that have allowed us to understand the crucial contribution of the active molecules present in this plant for their biological and therapeutic potentialities, the relevance of this knowledge goes beyond chemical features, as it is necessary to understand that due to the increased daily living standards of rural populations, decisions regarding the sustainable use of plant resources have been even more underlined [27, 28]. In this sense, this review aims to provide an overview of the botanical features, chemical composition, and biological effects of *Urtica* species towards well-being promotion and disease prevention.

## 2. Botanical Features and Geographical Location

*Urtica* species is a nitrophilous plant that can grow up to 1-2 m in height depending on edaphic conditions. Despite growing well in areas with high water availability [29–31], the plant can spread widely with its stoloniferous rhizomes [32].

Leaves are simple, dark green, stipulate, opposite, serrated, oblong, or ovate with cordate base [33, 34]. Both leaves surfaces are coated with stinging hairs; except in the European variety (*Urtica galeopsifolia*), the stinging hairs are absent [35]. Stem is green, erect, hollow to solid, fibrous and tough, indumentum of many stinging hairs and trichomes.

Flowers are small, reddish-brown to greenish-white in colour, mostly dioecious occurring as racemes in the axial of the upper leaves; staminate flowers with 4-5 long tepals, stamens 4, exserted, filaments flat; pistillate flowers with 4 short tepals, sparsely pubescent, esetulose, ovary superior, ovoid, 1-celled [1, 32, 34].

*Urtica* species present a subcosmopolitan distribution, being found around the globe, except in Antarctica and some tropical regions [36, 37]. The plant is commonly found as a weed, mainly in moist and shady places and often in anthropogenic habitats. The genus comprises 46 species, being the most important *Urtica dioica* (stinging nettle) and *Urtica urens* (small nettle), which are native to Europe, Africa, Asia, North America, and naturalized in other temperate parts of the world (Table 1) [2, 5].

The widely distributed weedy species, *U. dioica,* is considered an ecological keystone species and, thus, it is significantly important for the biodiversity in the ecosystem [38–42]. Island endemics are very common within this genus and the species include *U. dioica* subsp. *cypria* on Cyprus island, *Urtica atrovirens* on Corsica and Sardinia, *Urtica rupestris* on Sicily, *Urtica stachyoides* on the Canary Islands, *Urtica portosanctana* on Madeira, *Urtica bianorii* on Mallorca, *Urtica domingensis* on Hispaniola, *Urtica glomerulaeflora* on Juan Fernández Islands, *Urtica grandidentata* on Indonesia, *Urtica taiwaniana* on Taiwan, *Urtica papuana* on Papua New Guinea, and *Urtica perconfusa* on New Zealand. This indicates that island colonization within the genus is a unique feature amongst the flowering plants [3, 4].

## 3. Phytoconstituents

Phytochemicals are plant metabolites produced in response to any infectious attack or as a byproduct of any metabolic pathway, despite exerting beneficial effects in many ways [43, 44]. The active chemical part of nettle includes nearly fifty compounds of the lipophilic and hydrophilic fractions and whose chemical structure is known. Globally, few *Urtica* species have been screened for their phytochemical composition, with those available so far reporting the presence of sterols, triterpenes, coumarins, phenols, lignans, ceramides, and fatty acids, amongst other minor compounds, all with a distribution varying in the various organs of the plant (Tables 2–11).

Beta-sitosterol, transferulic acid, dotriacontane, erucic acid, ursolic acid, scopoletin, rutin, quercetin, and phydroxylbenzalcohol are some of the constituents found in *Urtica* species that may be applied for preventive or therapeutic purposes in communicable and noncommunicable diseases [16, 45–59]. The liquid contained in the hairs of a nettle causes it to sting, being composed of formic acid and leukotrienes in modest amounts, 1% acetylcholine, 1 in 500 to 1 in 2000 histamine, and 5-hydroxy-tryptamine (serotonin).

Essential ketones (38.5%), esters (14.7%), free alcohols (2%), nitrogenous compounds, phenols, aldehydes, p-sitosterol, formic acid and acetic acid, chlorophyll and phytol, vitamins, and carotenoids are also found in the aerial sections. Many organic acids were also identified in the aerial parts, including caffeic, ferulic, caffeylmalic, chlorogenic, and sinapic acids, according to chromatographic examination.

Flavonoids: isorhamnetol 3-O-glucoside, quercetol 3-O-glucoside, kaempferol 3-O-glucoside, isorhamnetol 3-O-rutinoside, and quercetol 3-O-rutinoside were extracted and identified in flowers, in addition to p-sitosterol, p-sitosterol glucoside, and scopoletol, which are found in all sections of the plant. The roots contained many molecules belonging to different chemical families, including polysaccharides: glycans, glucogalacturonans, arabinogalactan acid, fatty acid: (10E, 12Z)-9-hydroxy-10, 12-octadecadienoic acid, lectins, ceramides, terpenes diols, and terpenes diols glucosides [60].

Amongst *Urtica* species, *Urtica pilulifera* and *U. dioica* essential oil compositions have been investigated and consist mainly of hexahydrofarnesyl acetone, 1,8-cineole, *α* -ionone, *β*-ionone, farnesylacetone, methylbenzene, (−)-limonene, 3-carene, (+)-limonene, gamma-terpinene, vanillin, butyl acetate, 1, 2-benzenedicarboxylic acid, and 7-acetyl-6-ethyl-1, 1, 4, 4-tetramethyltetralin (Table 12) [61, 62]. Overall, considerably less attention has been paid to the phytochemistry of bioactive compounds in these plants.

## 4. Pharmacological Activities of the Genus *Urtica*

**Figure 1:**
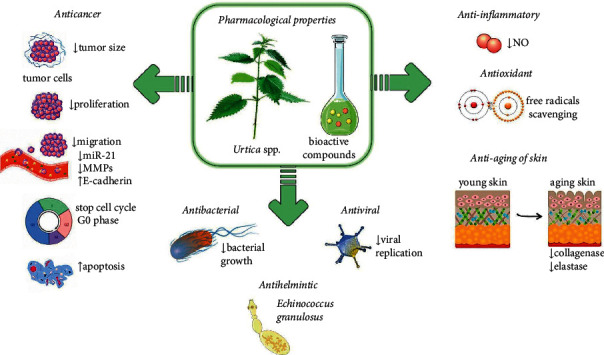
The most important pharmacological properties and potential mechanisms of bioactive compounds of *Urtica* spp. ↑: increase; ↓: decrease; NO: nitric oxide; MMPs: matrix metalloproteinases; miR-21: microRNA-21.

Except for *U. dioica,* which has extensively been studied for various pharmacological properties, few *Urtica* species have been investigated for their biological activity, including *U. angustifolia, U. laetivirens, U. parviflora, U. dentata, U. pilulifera, U. mairei, U. membranacea, U. urens, U. circularis, U. hyperborean, U. cannabina,* and *U. thunbergiana* that mostly displayed anti-inflammatory and antioxidant activities (Tables 1–13), and for antiviral, antimicrobial, antihelmintic, anticancer, nephroprotective, hepatoprotective, cardioprotective, antiarthritis, antidiabetic, antiendometriosis, and antiaging purposes (Figure 1)

### 4.1. *In Vitro* Pharmacological Findings

#### 4.1.1. Antiviral Activity

Antiviral treatment is limited to severe cases of most viral infections, stressing the need for more effective therapy. The aqueous extract of *U. dioica* fresh bark showed an antiviral effect against Petaluma virus (FIV-Pet) that infected Crandell feline kidney cell line (CrFK) by significantly inhibiting viral replication through reducing syncytia formation at low doses (0.5–1 g/ml) in a dose-dependent manner [73].

*U. dioica* extract (0.5–1 g/ml) and derived N-acetyl glucosamine-specific lectin (the 50% effective concentration (EC_50_) for HIV ranged from 0.3 to 9 mg/ml) also revealed to be able to inhibit syncytium synthesis between CD4+ MOLT/4 cells and HUT-78 cells when infected by HIV-1 and HIV-2 (Uncini Manganelli, Zaccaro & Tomei, 2005). Also, the N-acetyl glucosamine-specific lectin from *Urtica dioica* was inhibitory to cytomegalovirus (CMV), respiratory syncytial virus (RSV), and influenza A virus-induced cytopathic at an EC_50_ ranging from 0.3 to 9 mg/ml [74].

Another study showed that *U. dioica* agglutinin (UDA) suppressed the SARS-CoV virus replication by 90% at a concentration of 1.1 ± 0.4 ug/ml in Vero 76 cells by likely targeting the early stages of the replication phase through binding to the glycoprotein associated with the pseudotyped virus, thereby preventing the virus attachment to host cells [70].

#### 4.1.2. Antimicrobial and Antifungal Activity

Despite the growing number of antimicrobials available, the rate of microorganisms with acquired drug resistance is alarming, and thus more research is needed to discover alternative therapies more effective and safer than the currently available ones [75, 76].

*U. dioica* ethanol and aqueous extracts showed antibacterial activity against both Gram-positive and Gram-negative bacteria and yeasts, including *Proteus mirabilis, Pseudomonas aeruginosa, Enterobacter aerogenes, Escherichia coli, Citrobacter koseri, S. pneumonia, S. aureus, M. luteus, S. epidermidis*, and *Candida albicans.* They were also active against *M. tuberculosis* in case of multiple drug resistance [77–79]. Of note, the aqueous (microwave-assisted, ultrasound-assisted, and subcritical water extraction) and ethanol extracts of *U. dioica* leaves also confirmed antibacterial activity with minimal inhibitory concentration (MIC) of 9.76 ug/mL and 0.0625–0.500 mg/ml against methicillin-resistant (MRSA) and methicillin-sensitive (MSSA) *S. aureus* strains [80]; these observed effects were linked to their high content of hydroxycinnamic acids (chlorogenic, caffeic, and rosmarinic acids) and flavonoids (quercetin) (Table 1) [81].

#### 4.1.3. Anthelmintic Activity

The ethanolic extract of *U. dioica* displayed *in vitro* anthelmintic activity against protoscoleces of *Echinococcus granulosus*, increasing the concentration and duration of exposure, reaching 96.2% inhibition at a concentration of 4 *μ*g/ml for 30 min (Table 1) [82]. Anthelmintic activity of the methanol extract was also investigated using adult Indian earthworms (*Pheretima posthuma*) and revealed a dose-dependent increase in anthelmintic activity at 25, 50, and 100 mg/mL [83].

#### 4.1.4. Anticancer Activity

Cancer is the largest cause of death in the world due to poor timely access to high-quality diagnosis and treatment [84, 85]. *U. dioica* significantly suppressed the human breast cancer cell line (MCF-7) and fibroblasts secluded from foreskin tissue, with IC_50_ values of MCF-7 (31.37 mg/ml), MDA-MB-23 (38.14 mg/ml), 4T1 (44.07 *μ*g/mL to 35.21 mg/ml), and HFFF2 (69.42 mg/ml). QRT-PCR showed that *U. dioica* extracts inhibited cell migration by downregulating the expression of miR-21, matrix metalloproteinase (MMP) 1, MMP9, and MMP13, and C-X-C motif chemokine receptor 4 (CXCR4) and upregulating the expression of E-cadherin [86]. *U. dioica* leaves also increased cell apoptosis in 4T1 cells [72].

The aqueous extract of *U. dioica* leaves significantly decreased the cell proliferation of AML U937 cell line (acute myeloid leukemia), with IC_50_ of 24 ug/ml for the first 48 h and then 16 ug/ml after 72 h [87]. Moreover, flow cytometry showed that the extract was able to stop the cell cycle into the G0 phase and increase cell apoptosis at the early and late stages by increasing proapoptotic protein Bax expression and decreasing antiapoptotic protein Bcl-2 expression [87].

Zekovic et al. reported the antiproliferative effect of the subcritical water extract of *U. dioica* against Hep2c, RD, and L2OB cells (13.42 ug/ml, 9.69 ug/ml, and 7.52 ug/ml, respectively) (Table 1) [80]. Moreover, the bioactive compound, 5a, 6b-dihydroxy-daucosterol, from *U. laetevirens* showed anticancer activity against MH7A cells by inhibiting proliferation and inducing apoptosis (Table 1) [88, 89].

#### 4.1.5. Antioxidant Activity

Antioxidants are synthetic or natural compounds that can help to prevent or delay cell damage [90, 91]. The aqueous extract of *U. dioica* leaves presented antioxidant activity, assessed through the DPPH radical scavenging (IC_50_ = 16.93 ug/mL), reducing power (EC_50_ = 30.07 ug/mL) and polarographic (HPMC = 243.2%/mL) assays [80].

Batches of *U. dioica* analyzed for their antioxidant potency revealed batch 14 as the most potent (2.71 TEAC) using the CUPRAC assay and batch 27 (0.73 TEAC) using the FRAP assay. The resulting response surface plots approved a positive association between the antioxidant actions and the phenolic acids content [92]. A comparative study performed by Carvalho et al. demonstrated the superior antioxidant properties of *U. dioica* in all assays: DPPH (2.89 g/100 g lyophilized), ABTS (2.60 TEAC), and FRAP (3.81 TEAC) when compared to *U. membranacea* and *U. urens* aerial parts (Table 1) [89].

Methanol and direct-ethanol extracts of *Urtica* root showed free radical scavenging activity of 46.71% and 45.03% at 500 *μ*g/ml, respectively. Moreover, *Urtica parviflora* (methanol/aqueous extract) has been reported for free radical scavenging and reducing activity, with biological activity varying in a dose-dependent manner. The antioxidant potential has also been reported in the ethanolic extracts of *Urtica circularis, Urtica hyperborean* (methanol extract), *Urtica cannabina* (polyphenols), and *U. urens* (Table 1) [93–96].

#### 4.1.6. Anti-Inflammatory Activity

Although nonsteroidal medicines can be useful, herbs can be a safer and often effective alternative for pain management, especially when used for a long period [97]. *U. dioica* (leaves extract) and isolated flavonoids were active against thrombin-induced platelet aggregation (IC_50_ values of 0.25 ± 0.05 and 0.40 ± 0.04 mg/ml) [98]. A comparative study between 50% ethanol extracts of *U. dioica*, *U. membranacea*, and *U. urens* aerial parts showed that *U. urens* extract (350 ug/mL) could act as a more potent anti-inflammatory agent by showing the highest reduction in nitric oxide production (up to 41%) (Table 1) [89].

#### 4.1.7. Antiaging of Skin

Those who are prone to wrinkles and fine lines and those who have loose, sagging skin usually consider antiaging therapies [99]. Different extracts of *U. dioica* demonstrated antiaging efficacy using elastase and collagenase enzymes inhibition assay. The more potent batches were batch 1 that inhibited collagenase enzyme by 16.23% and batch 26 that inhibited elastase enzyme by 24.51%. This potency was linked to the high content of quercetin and ursolic acid, respectively (Table 1) [92].

### 4.2. *In Vivo* Pharmacological Findings

#### 4.2.1. Antiviral Activity

*U. diorca* was also investigated for its *in vivo* antiviral potency. *U. dioica* agglutinin (UDA) at a dose of 5 mg/kg (b.w/day; i.p.) significantly sheltered the mice against lethal infection with the virus but did not decrease the virus titers in the lung of the SARS-CoV-infected BALB/c mouse model, also preventing the weight loss and lung pathology scores of infected mice [70] (Table 13).

#### 4.2.2. Anthelmintic Activity

The fight against helminth infectious is still pending complete eradication either through a vaccine or pharmacological therapies. *In vivo* study showed that daily oral administration (175 mg/ml) of the methanol extract obtained from leaves and seeds of *U. dioica* showed anthelmintic activity in Swiss albino mice naturally infected with *Aspiculuris tetraptera* (Table 13) [100].

#### 4.2.3. Anticancer Activity

The dichloromethane extract of *U. dioica* further showed anticancer activity by significantly reducing the tumor size and weight on 4T1 (breast cancer cell line) allograft tumor in BALB/c mouse model at 10 and 20 mg/kg b.w/day (i.p.). This efficacy was linked to increased cell apoptosis and suppression of cell proliferation through BCL2 downregulation and increased caspase-3 activity [72].

#### 4.2.4. Nephroprotective

The kidney is a key organ of the metabolism of any xenobiotic; thus, preventing its alteration is crucial [101]. The 95% ethanol extract of *U. dioica* showed therapeutic action against nephrotoxicity on gentamicin-induced nephrotoxicity in the male rabbit model at a dose (100 mg/kg b.wt./day P.O.). The extract has a potent antioxidant activity through enhancing glutathione level and decreasing malondialdehyde level and helps in controlling serum creatinine and blood urea nitrogen levels [69]. *Urtica parviflora* extract (aerial parts) showed neuroprotective activity against nephrotoxicity induced by paracetamol and gentamicin and renal disability in Wistar rats and rabbits (Table 1) [77, 96].

#### 4.2.5. Hepatoprotective

Because the liver is such an important part of any xenobiotic metabolism, preventing its alteration is also of utmost importance [102]. *U. urens* and *U. dioica* have been reported for their hepatoprotective activity against CCl4-induced liver toxicity in rats. For example, *U. dioica* (methanol extract) promoted an antioxidant system against cisplatin-induced toxicity in Ehrlich ascites tumor (mice model) and exerted hepatoprotective activity (Table 13) [83, 89].

#### 4.2.6. Cardioprotective

Cardioprotection refers to all systems and methods that help keep the heart healthy by decreasing or even preventing myocardial damage [103]. *U. dioica* water and petroleum ether extract at, respectively, 20 and 150 mg/kg/day improved blood lipid level in rats, decreased blood cholesterol levels and LDL/HDL lipoprotein ratios after 30 days. On the other hand, *U. dioica* ethanol extract decreased cholesterol and LDL levels at a dose of 100 and 300 mg/kg [104, 105]. *Urtica parviflora* (350 and 500 mg/kg p.o.) effectively decreased cardiac complications and enhanced serum LDL level.

*U. dioica* aqueous extract (1 and 2 g/L) decreased heart rate and improved pressure in the left ventricle in Langendorff-perfused rat heart. It also improved the tolerance level of isolated rat heart against ischemia-reperfusion (Table 13) [106–108].

#### 4.2.7. Antiarthritis Effect

*Urtica* species has also been shown to be effective for anti-inflammatory purposes, particularly in the treatment of arthritis. For example, a total coumarins extract from *Urtica dentata* demonstrated a dose-dependent antiarthritis activity in collagen-induced arthritis BALB /c mice model at three doses (20, 40, and 60 mg/kg b.w. P.O. every other day). Total coumarins also protected tissues against bone destruction by reducing IFN-g and IL-2 production and increasing IL-10 and TGF-B (Table 13) [63].

#### 4.2.8. Antidiabetic Effect

Diabetes mellitus is a significant metabolic illness that can affect the central nervous system in a variety of ways, both functionally and morphologically [109]. Ethyl acetate and chloroform extracts of *U. pilulifera* showed antidiabetic activity at two doses (250 and 500 mg/kg b.w./day P.O.) on streptozotocin and high-fat diet-induced type 2 diabetes adult male albino rat model. Briefly, the extracts decreased glucose level, HbA1C percentage, and insulin resistance, with this hypoglycemic effect being associated with the anti-inflammatory effect through reducing C-reactive protein (CRP) levels in serum and TNF-a level and exerting antioxidant activity through decreasing MDA and increasing GSH levels, SOD, and catalase activities in pancreatic tissues (Table 13) [65]. Also, a formulation containing *U. dioica, Artemisia judaica, Morus folium, Taraxacum officinale,* and *Canella winteriana* has been reported to treat insulin-dependent (type I) and noninsulin-dependent (type II) diabetes. Furthermore, a lectin isolated from seeds of *U. pilulifera* exerted an antidiabetic impact on diabetic rats (streptozotocin (STZ) model) when administered for 30 days at a dose of 100 mg/kg. *U. parviflora* leaves (aqueous extract) also exerted hypoglycemic effect in normoglycemic rats, while *U. angustifolia* (leaves, stems, and roots) exerted hypoglycemic effects in a dose-dependent way (Table 13) [79, 110–112]. More recently, ZnO nanoparticles of aqueous extract from *U. dioica* leaves confirmed the antidiabetic activity of the combination (8 mg/dl. b.w./day I.P.) in an alloxan-induced diabetic rat model by significantly decreasing fasting blood glucose, total cholesterol, and total triglycerides levels in serum, while increasing high-density lipoprotein and insulin levels (Table 13) [66].

#### 4.2.9. Antiendometriosis Effect

Endometriosis is a painful disorder in which tissue from the womb's lining (uterus) is present both inside and outside the uterus. Some herbs may raise the risk of endometriosis, while others may help to heal it faster. The methanol extract of *U. dioica* aerial parts showed an antiendometriosis effect on the surgery-induced endometriosis rat model at a dose of 100 mg/kg b.wt./day P.O. by decreasing implant volumes and adhesion scores and peritoneal TNF-*α*, VEGF, and IL-6 levels as supported by histopathological outcomes (Table 13) [67].

#### 4.2.10. Effect on Prostate Hyperplasia

Prostate enlargement, commonly known as benign prostatic hyperplasia (BPH), is a noncancerous increase in the size of the prostate gland. Prostatic hyperplasia was suppressed by a polysaccharide fraction of *Urtica* on testosterone propionate-induced prostate hyperplasia castrated rat model at three doses (62.5, 125, and 250 mg/kg b.wt. P.O.). Treatment with the lowest dose (62.5 mg/kg) reduced the indexes of wet weight, dry weight, and volume by 17%, 23%, and 32%, respectively. With the highest dose (250 mg/kg), the indexes of wet weight, dry weight, and volume were further reduced by 25%, 33%, and 37%, respectively (Table 13) [68].

Many herbal preparations from *U. dioica* extracts can inhibit 5-*α*-reductase [113]. Indeed, *U. dioica* roots (methanol extracts) were able to inhibit aromatase (AR) and 5a-reductase (5aRE) in a dose-dependent manner (ED_50_ of 3.58 and 14.7 mg/mL, respectively). *Urtica mairei* (roots) reduced BPH and inhibited the activity of 5a-reductase (Table 13) [114, 115].

Hartmann et al. evaluated the effect of a combination between methylene chloride extract of *Pygeum africanum* bark and 30% methanol extract of *U. dioica* roots with a ratio of 1 : 12 (Prostatonin®) on BPH. This combination also significantly inhibited reductase and aromatase enzymes with ED_50_ of 14.15 mg/ml and 0.24 mg/ml, respectively (Table 13) [116].

#### 4.2.11. Antioxidant Activity

Antioxidants are widespread in the plant kingdom. For example, the 80% ethanol extract of *U. dioica* leaves confirmed antioxidant activity in a normal Swiss albino mouse model at two doses (50 and 100 mg/kg b.w./day P.O.). Both doses of the extract led to a marked increase in the activities of cytochrome b5, NADH-cytochrome b5 reductase, glutathione S-transferase, DT-diaphorase, glutathione peroxidase, glutathione reductase, superoxide dismutase, and catalase in liver tissues. On the other hand, they showed a reduction in cytochrome P450, lactate dehydrogenase, NADPH-cytochrome P450 reductase, total sulfhydryl groups, nonprotein sulfhydryl groups, and protein-bound sulfhydryl groups (Table 13) [64].

#### 4.2.12. Anti-Inflammatory Activity

The discovery of new anti-inflammatory agents has long been a source of concern. The aqueous extract of *U. dioica* leaves showed analgesic effect at 1200 mg/kg by reducing thermal situation in a hot plate test (55°C), improving resistance to ache and hyperstimulation of the sensory nociceptors leading to TENS-like effect [94, 117].

The aerial part of *U. urens* (ethanol extract) inhibited 62.8% of the licking time during the final stage of the formalin test at a dose of 500 mg/kg in chemically induced mouse pain models [118, 119]. *U. urens* (methanol extract of aerial parts) at 100 to 400 mg/kg significantly displayed anxiolytic effect against mice model (Table 1) [120].

Furthermore, *U. dioica* aqueous extract (150 mg/kg dose) showed antipyretic activity in albino mice, while *Urtica macrorrhiza* aqueous extract (stem) decreased fever intensity in rats at 200 and 400 mg/kg [79, 121]. Indeed, it has been reported that *U. dioica* act by either blocking or interfering with chemical processes in the body related to chemicals found in the body, including dihydrotestosterone. In the carrageenan-induced paw edema model of rats, *U. urens* showed outstanding anti-inflammatory efficacy. Extract of its aerial parts revealed a percentage inhibition of 41.5% at 300 mg/kg i.p. in case of hind paw edema in rats. Moreover, petroleum ether extract of seeds of *U. pilulifera* and n-butanol and aqueous of *U. macrorrhiza* have also reported anti-inflammatory activity against carrageenan-induced paw edema in rats (Table 13) [111, 120, 122, 123]. In addition, some compounds from *Urtica circularis*, namely vicenin-2, caffeic acid, chlorogenic acid, and vitexin displayed a dose-dependent antinociceptive activity in nociceptive mice, in the following order of activity: vitexin (91%)> caffeic acid (41%) = vicenin-2 (41%)> chlorogenic acid (72%) (Table 13) [118, 119].

#### 4.2.13. Antiaging of Skin

The use of natural plant extracts in the cosmetic industry as antiaging agents has received rising attention. Hwang et al. demonstrated the antiaging activity of the 50% ethanol extract of *Urtica thunbergiana* leaves on UVB-induced skin aging hairless mouse model at two doses (0.1% and 1% g/kg b.w. of animals' diet). The extract (100 ug/mL) improved the aging disorders implied by UVB-irradiated NHDF, with ROS generation being reduced by 17%, MMP-1 and MMP-3 by 61% and 29%, respectively, and IL-6 secretion by 60%. Moreover, procollagen type 1 generation was upregulated by 255% and phosphorylation of ERK, JNK, and p38K was suppressed by 14%, 32%, and 38%, respectively. Dephosphorylation of NFAT was also inverted possibly due to the high content of chlorogenic acid in *U. thunbergiana* (Table 13) [71].

#### 4.2.14. Diuretic and Antiurolithiatic Effects

*U. dioica* has traditionally been used as a diuretic in indigenous medicine. Experimentally*, U. dioica* (aqueous extract) possess natriuretic and diuretic activity in rabbits; the rate of K^+^ remains unaffected. *U. dioica* also revealed effectiveness against urinary infections. Indeed, its aerial part (methanol extract) also exerts antiurolithiatic potential can suppress the increased levels of urinary calcium and creatinine while significantly reducing the renal deposition of calcium and oxalate. *U. dentata* (n-butanol extract) also exert antiurolithiatic activity, prevent the deposition of calcium oxalate, and protect renal tissue from injury produced by kidney calculi (rat model) (Table 13) [112, 124].

### 4.3. Miscellaneous

The hypotensive activity of the methanol and water extract of *U. dioica* has also been shown in human cells culture and *in vitro* models of prostatic antihyperplasic activity [125]. *U. dioica* aqueous extract has been revealed to exert good *in vivo* antiulcer efficacy against ethanol-induced ulcers [113], while leaves and seed extract (400 *µ*g/mL) possess *in vitro* immunomodulatory potential (Table 13) [47, 126]. Finally, *U. angustifolia* (polysaccharides) showed antifatigue properties in mice [112].

## 5. Health-Promoting Effects: Clinical Trial Findings

### 5.1. Anti-Inflammatory Effect

Earlier literature reported that the administration of 1340 mg of powdered extract of *U. dioica* (nettle leaves) reduced arthritis to half. A randomized control trial in 50 patients suffering from a chronic joint disease in Germany demonstrated the effectiveness of a combination of stewed nettle along with 50 mg of diclofenac treatment (group D50+U) compared to a standard dose of diclofenac (200 mg) [127, 128]. Results of this study indicated that both treatments were equally effective in mitigating clinical symptoms occurring due to acute arthritis. These results are of great importance for patients who suffer from nonsteroidal anti-inflammatory drugs (NSAIDs) intolerance because of ulceration or other gastric problems. However, further studies are required to find out whether nettle could be effective in the absence of NSAIDs [127, 129].

### 5.2. Diuretic Effect

In a study aiming to assess the impact of 15 mL nettle herb juice for treating myocardial or chronic insufficiency, 32 patients received 3 times daily such preparation in an open 2-week study. Later, the frequency of dosing was reduced to once a day in the morning. The daily volume of urine was increased significantly throughout the treatment. The patients with myocardial insufficiency in the 2^nd^ day of treatment was 9.2% higher (*p* ≤ 0.0005) than the baseline and patients of chronic venous insufficiency reported 23.9% higher (*p* ≤ 0.05) urine volume. Patients' weight (about 1%) and systolic blood pressure showed slight decreases. Apart from slight side effects, like diarrhea, serum parameters remained stable and treatments were smoothly tolerated. Additionally, diuretic and natriuretic effects were detected, implying a renal function effect [127, 130]. Some objective indicators in this clinical investigation indicated statistically significant improvement, despite the small number of patients and the short duration of the study limiting the establishment of solid conclusions.

### 5.3. Antiallergic Effect

The safest remedy for allergy and sinus treatment is nettle. Indeed, it has been reported used in various ailments ranging from allergic rhinitis to hypertension. Lyophilized leaves of nettle have been clinically proven to relieve allergy symptoms [89, 113]. For example, a double-blind, randomized study was conducted with 98 individuals to try the effect of freeze-dried *U. dioica* herb (2 times 300 mg) on allergic rhinitis. After one week of therapy, daily symptom diaries and global response documented after follow-up were considered for assessment [131, 132]. In the overall evaluations, *U. dioica* was ranked higher than placebo, and when the diary data were compared, *U. dioica* was just marginally higher [132]. Thus, even if the *U. dioica* trial appears to be effective, more research with a bigger and better-matched sample size and possibly a longer treatment period might be beneficial. Research into the mechanism of action of *U. dioica* and its potential for application in other allergy disorders is also recommended.

### 5.4. Antidiabetic Effect

The health benefit of the hydroalcoholic extract of *U. dioica* on blood lipids, hepatic enzymes, and nitric oxide levels was investigated in a randomized control trial, including 50 women with type 2 diabetes. *U. dioica* significantly decreased FPG and TG and increased SGPT levels and HDL, NO, and SOD levels compared to the control group after 8 weeks of treatment. This result supports using the hydroalcoholic extract of *U. dioica* as an antioxidant agent for additional therapy of diabetes to minimize complications, such as cardiovascular risk factors in diabetic patients [133]. However, the relatively small sample size and the lack of exact diet and exercise management of patients who participated in the study make the findings suggestive rather than conclusive. Therefore, trials with a larger number of patients and a longer intervention period are recommended to better understand *U. dioica*'s benefits in diabetic patients.

Overall, these clinical studies are not appropriate for traditional use in indications, like the acute attack of chronic joint disease, myocardial or chronic venous insufficiency, and allergic rhinitis. Indeed, only the well-established use can be relevant in these indications; however, they are hardly good enough and the results of these trials cannot be used [134].

Since a small number of participants were included in the studies and were not double-blind (except Mittman's study) and that data are not detailed enough, the consequences are not influential. These studies may only support the authenticity of diuretic and anti-inflammatory effects; in this way, the traditional indications may be supported by them.

## 6. Safety, Drug-Drug Interaction, and Adverse Effect of the Genus Urtica

Though sweating and gastric discomfort are reported in some cases, the *Urtica* plant usually causes skin irritation upon touching it [135].

Hypersensitivity cases have been reported in patients with renal ailments [33, 130]. When the hairs or spines on the stems and leaves of the stinging nettle come into contact with the skin, various physiologically active chemicals are released within seconds and in turn induce irritation, dermatitis, and urticaria [136]. These findings imply that histamine, which is released by the nettle, has a role in the rapid reaction to nettle stings. Moreover, the endurance of the stinging sensation, on the other hand, could indicate that there are chemicals in nettle fluid that are directly harmful to nerves or that can cause the subsequent release of other mediators [137]. Furthermore, urine flow is enhanced by the aerial parts of *Urtica*; hence, it is advised to inform the healthcare provider of whether the patient suffers from diabetes or kidney problems [33, 130]. *Urtica* aerial parts at 1.25 g/kg decline blood sugar following intake [135] and may potentiate concurrent antidiabetics' effect, high or low blood pressure [138–140].

Furthermore, the key underlying processes of this food plant and its phytonutrients in the management of urolithiasis include a diuretic effect, which can exacerbate the diuretic therapy in patients with renal disorders. Though nettle is reputed to be an abortifacient and to affect the menstrual cycle in traditional medicine, oral administration of 250 mg/kg of nettle to mice is devoid of antifertility activity. In the absence of clear evidence of antifertility potency, *Urtica* spp. should be completely avoided during pregnancy or in breastfeeding women and children [127]. For sure, *Urtica dioica* and *Urtica urens* preparations have been used orally as a postpartum “tonic” for treating anemia in nursing mothers and is a purported galactagogue. Still, no scientifically valid clinical trials support the safety and efficacy in nursing mothers or infants for any use [141].

*Urtica dioica* is used as an anti-inflammatory in rheumatoid arthritis. The anti-inflammatory effect of *Urtica* extract is due to its inhibitory effect on NF-kappaB activation and the genetic transcription factor that activates TNF-*α* and IL-1B in synovial tissue that lines the joint, lowering TNF-*α* and other inflammatory cytokines levels [123, 142]. Therefore, *Urtica* spp. should be avoided in the case of acute arthritis due to the risk of drug-drug interaction [138].

*Urtica* spp. has also been reported to enhance the impact of CNS depressant medications [138]. The concomitant use of *Urtica* aerial parts with sedatives, including lorazepam (Ativan), phenobarbital (Donnatal), clonazepam (Klonopin), zolpidem (Ambien), and others may lead to sleepiness and drowsiness [127].

## 7. Conclusions and Future Perspectives

In short, while summarizing the ethnopharmacological reports on the use of *Urtica* species, *U. dioica* emerged as the most reported species, providing a rich source of active principles for developing novel treatment strategies. Despite its ancient use by people from different cultures and in different regions for the treatment of various ailments, the current achievements have stated that *Urtica* spp. have renowned pharmacological potentialities, including anti-inflammatory, anticancer, antioxidant, antidiabetic, antimicrobial, and antiviral effects that correlate, by one hand, with some traditional uses and, on the other hand, with the bioactive phytochemicals present, including phenolic compounds and terpenoids that may be effectively applied for preventive or therapeutic purposes in communicable and noncommunicable diseases. However, there is still a large gap in *in vivo* experiments and clinical trials using plant-based preparations or isolated phytochemicals from *Urtica* spp. that need to be filled in a short time so that new windows for preventive, therapeutic, and agroindustrial purposes can be open.

## Figures and Tables

**Table 1:** Geographical distribution, traditional uses, and pharmacology of *Urtica* species.

No.	Species	Geographical distribution	Traditional uses	Pharmacological activities
1	*Urtica andicola* Wedd.	Turkey	Skin rashes, arthritis, fungal infections	—
2	*Urtica angustifolia* Fisch. ex Hornem.	China, Japan, Korea, Mongolia, Siberia	None known	Antifatigue
3	*Urtica ardens* Link	Bhutan, India, Nepal, Sikkim	Exorcism, jaundice, postcalving care, sprains, bones fracture, hematuria, neck sore, yolk sore	—
4	*Urtica aspera* Petrie	New Zealand	Stomach diseases, snakebites, inflammation, rheumatoid arthritis, hyperplasia, fungal infections	—
5	*Urtica atrichocaulis* (Hand.-Mazz.) C.J. Chen	China, Japan, Korea, Himalayas, Pakistan	Rheumatoid arthritis, inflammatory, antioxidant, immune-modulatory	—
6	*Urtica atrovirens* Req. ex Loisel	France, Italy, Spain	Antihyperglycemic, antioxidant, hepatic protective, antiviral, arthritis	—
7	*Urtica australis* Hook.f.	New Zealand	Skin diseases, diabetes, eczema, fungal infections, arthritis	—
8	*Urtica ballotifolia* Wedd.	Colombia, Ecuador	—	—
9	*Urtica berteroana* Phil	Chile, Bolivia, Argentina, Colombia	—	—
10	*Urtica buchtienii* Ross	Chile, Argentina	—	—
11	*Urtica cannabina* L.	Russia, Sweden, Netherlands, China, Western Asia from Siberia to Iran	—	Anti-inflammatory
12	*Urtica chamaedryoides* Pursh	United States, Mexico	—	—
13	*Urtica circularis* Sorarú	Brazil, Argentina, Paraguay, Uruguay	—	Antioxidant, anti-inflammatory
14	*Urtica deltoidea* Sw.	New Zealand	Arthritis, inflammation, antiulcer, anticancer, antimicrobial activities	—
15	*Urtica dentate* Hand.-Mazz	North America	Kidney problems, rheumatoid arthritis, kidney calculi	Antiarthritis, antiurolithiatic
16	*Urtica dioica* L.	United States, New Zealand, Turkey, Europe, Asia, North America	Injuries to reduce swelling, diuretic, flu, diabetes disease, losing weight, cold, cancers, anemic conditions, libido, induce menstruation, stomach- ache, renal and pulmonary diseases	Antiviral, antimicrobial, antioxidant, anti-inflammatory antiaging, cytotoxic/anticancer Effect on benign prostatic hyperplasia, antidiabetic, antiendometriosis, nephroprotective
17	*Urtica echinata* Benth	Bolivia, Peru, Argentina, Ecuador	—	—
18	*Urtica ferox* Blanco	New Zealand, Australia	Skin problems, hyperglycemic, antiviral, diuretic, hypotensive, antiaggregate	—
19	*Urtica fissa* E. Pritz	China, Taiwan, Egypt, Vietnam	Rheumatoid arthritis	—
20	*Urtica flabellata* Kunth	Bolivia, Peru, Ecuador, Chile, Colombia, Turkey	Skin rashes, arthritis, fungal infections	—
21	*Urtica galeopsifolia* J. Jacq. ex Blume	Russia, Ukraine, Belarus	Renal ailments, asthma, anemia, blood purification	—
22	*Urtica gracilenta* Greene		Kidney diseases, diabetes, fungal infections	—
23	*Urtica glomeruliflora* Steud.	Chile		—
24	*Urtica haussknechtii* Boiss.	Turkey		—
25	*Urtica hyperborea* Jacq. exWedd.	Nepal, India, China	Skin rashes, arthritis, fungal infections	Antioxidant
26	*Urtica incana* Blume	Peru	Skin rashes, arthritis, fungal infections	—
27	*Urtica kioviensis* Rogow.	Europe, Israel, Russia	Arthritis, hepatic protective, antiviral	—
28	*Urtica lalibertadensis* Weigend	Peru	Skin rashes, arthritis, fungal infections	—
29	*Urtica laetevirens* Maxim.	China, Japan, Korea		Anticancer
30	*Urtica leptophylla* Kunth	Costa Rica, Colombia, Peru, Bolivia, Ecuador	Skin rashes, arthritis, fungal infections	—
31	*Urtica lilloi* (Hauman) Geltman	Argentina		—
32	*Urtica longispica* Killip	Ecuador, Peru, Colombia	Cough, eczema, gout, *urticaria*, allergic rhinitis, rheumatoid arthritis	—
33	*Urtica macbridei* Killip	Ecuador, Peru		—
34	*Urtica magellanica* Juss. ex Poir.	Chile, Peru, Bolivia, Argentina, Ecuador	Allergy, arthritis	—
35	*Urtica mairei* H. Lév.	China, India, Bhutan, Himalaya, Myanmar	Kidney pain. Its extract and paste kidney diseases, diabetes, fungal infections, inflammation, arthritis	Antiprotatic hyperplasia
36	*Urtica masafuerae* Phil.	Chile		—
37	*Urtica massaica* Mildbr.	Africa	Skin rashes, malaria, eczema, skin rashes, dermatitis, diuretic	—
38	*Urtica membranacea* Poir. ex Savigny	Israeli, Europe, Algeria	—	Antioxidant, anti-inflammatory
39	*Urtica mexicana* Liebm	Mexico, Guatemala	—	—
40	*Urtica mollis* Steud.	Peru, Chile, Argentina	—	—
41	*Urtica morifolia* Poir.	Europe	—	—
42	*Urtica orizabae* Liebm.	Mexico, United States, Cuba	—	—
43	*Urtica parviflora* Roxb.	Nepal, India, United States, Western China, Bhutan, Himalaya	Arthritis, tumor, astringent, diuretic, inflammatory	Nephroprotective, antidiabetic, antioxidant
44	*Urtica pilulifera* L.	Tunisia, Israel, Cyprus, Costa Rica, Turkey, Palestine	Skin and prostate disorders, rheumatoid arthritis, diabetes, skin treatment, inflammation, arthritis, internal bleeding, anemia, excessive menstruation, hemorrhoids, rheumatism, hay fever, kidney problems, pain, skin problems, abdominal pain, internal diseases, antiasthmatic, antitumor, astringent, diuretic, galactagogue, depurative, antihyperglycemic, antidandruff	Antidiabetic
45	*Urtica platyphylla* Wedd.	Japan, Russia	—	—
46	*Urtica praetermissa* V.W. Steinm.	Mexico	—	—
47	*Urtica pubescens* Ledeb.	Mexico	—	—
48	*Urtica rupestris* Guss.	Italy	—	—
49	*Urtica sondenii* (Simmons) Avrorin ex Geltman	Canada	—	—
50	*Urtica spiralis* Blume	Mexico	—	—
51	*Urtica stachyoides*Webb & Benth.	Spain, Mexico	—	—
52	*Urtica taiwaniana* S.S. Ying	Taiwan	—	—
53	*Urtica thunbergiana* Siebold & Zucc.	Japan, Korea, China	—	Antiaging
54	*Urtica triangularis* Hand.-Mazz.	China	—	—
55	*Urtica trichantha* (Wedd.) Acevedo & Navas	Chile, Bolivia, Peru, Japan, China	—	—
56	*Urtica urens* L.	Unite States, Mexico, Europe, Israel, New Zealand	Blood depurative, antihypoglycemic, antioxidant, hepatic protective, antiviral, diuretic, hypotensive, antiaggregate, kidney problems	Antioxidant, anti-inflammatory

**Table 2:** Lignans extracted from *Urtica.*

Sr. no.	Compound name	Structural
1.	Cycloolivil; 9′-O-b-d-Glucopyranoside	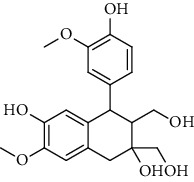

2.	4-[Bis(3, 4-dihydroxyphenyl) methyl]dihydro-3-(hydroxymethyl)-2(3H)-furanone; (8R^*∗*^, 8′R^*∗*^)-form,3′, 4-Di-Me ether, 7-O-b-D-glucopyranoside	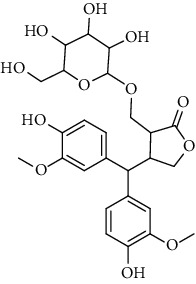

3.	4-[Bis(3, 4-dihydroxyphenyl) methyl]dihydro-3-(hydroxymethyl)-2(3H)-furanone; (8R^*∗*^, 8′R^*∗*^)-form,3′, 4-Di-Me ether, 4′-O-b-D-glucopyranoside	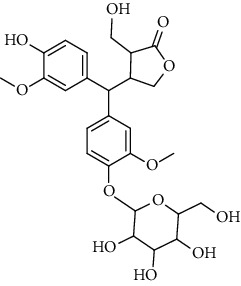
4.	4-[Bis(3, 4-dihydroxyphenyl) methyl]dihydro-3-(hydroxymethyl)-2(3H)-furanone; (8R^*∗*^, 8′R^*∗*^)-form,3′, 4-Di-Me ether	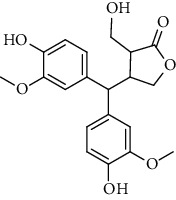

5.	Neoolivil	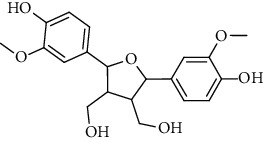

6.	3, 3′, 4, 4′, 8′, 9-Hexahydroxy-7, 9′-epoxylignan; (7S,8R, 8′S)-form, 3, 3′-Di-Me ether, 9-O-*β*-D-glucopyranoside	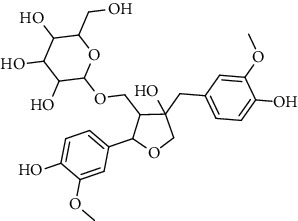

7.	3, 3′, 4, 4′, 8′, 9-Hexahydroxy-7, 9′-epoxylignan; (7S,8R,8′S)-form, 3, 3′, 4-Tri-Me ether, 8′-Ac	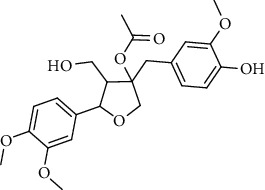
8.	3, 3′, 4, 4′, 8′, 9-Hexahydroxy-7, 9′-epoxylignan; (7S,8R,8′S)-form, 3, 3′, 4-Tri-Me ether, 8′-Ac, 4′-O-[aarabinopyranosyl-(1 ⟶ 6)-bd-glucopyranoside]	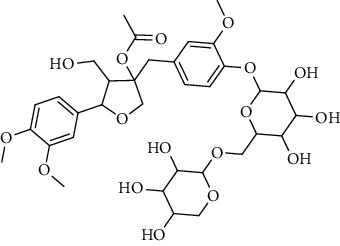

9.	Neoolivil; 9-Ac, 4-*O*-b-D-glucopyranoside	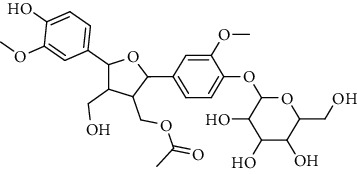

10.	Neoolivil; 4-*O*-b-D-glucopyranoside	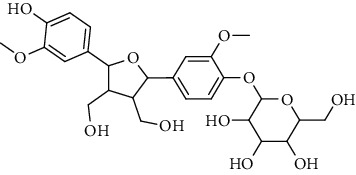

11.	Neoolivil; 9, 9′-Di-Ac, 4-*O*-b-D-glucopyranoside	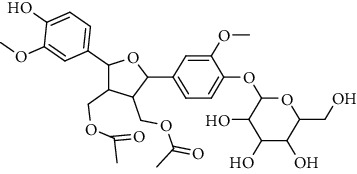
12.	Pinoresinol; (+)-form, 4-O-[a-L-rhamnopyranosyl-(1 ⟶ 2)-b-d-glucopyranoside]	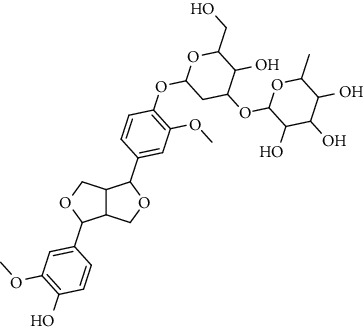

13.	Secoisolariciresinol	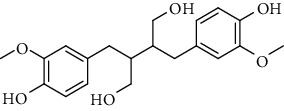

14.	Isolariciresinol	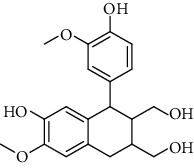

15.	Urticene; (−)-form	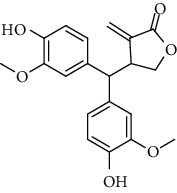
16.	Neoolivil; 9-O-b-d-Glucopyranoside	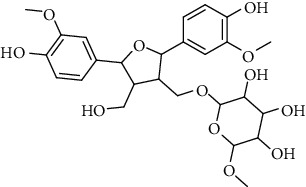

17.	Dehydrodiconiferyl alcohol	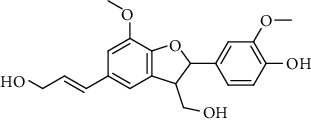

18.	Olivil	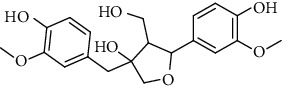

19.	3,4-Divanillyltetrahydrofuran	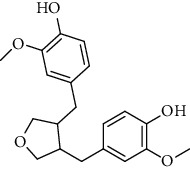

**Table 3:** Sterols extracted from *Urtica.*

Sr. no.	Compound name	Structural formula
1.	Stigmastane-3, 6-diol; (3*β*, 24R)-form, O-[b-d-Glucopyranosyl-(1 ⟶ 4)-al-arabinopyranoside]	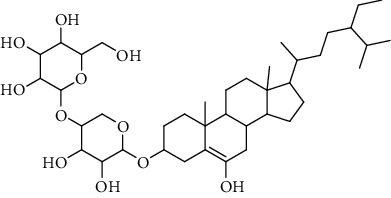
2.	Stigmastane-3, 6-diol; (3b, 7a, 24R)-form, 3-O-b-d-Glucopyranoside	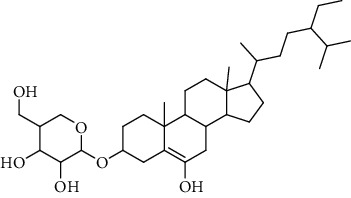
3.	Stigmastane-3, 6-diol; (3b, a6a, 24R)-form	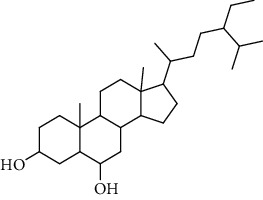
4.	Daucosterol	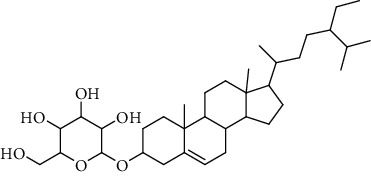
5.	Ethyl iso-allocholate	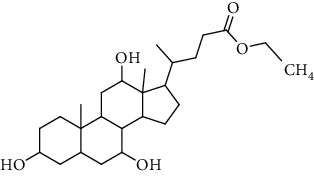
6.	Cholesterol	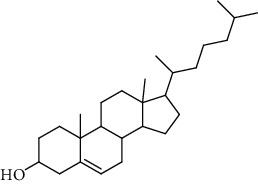

**Table 4:** Fatty acids isolated from genus *Urtica.*

Sr.	Name	Structural formula
1	Palmitic acid	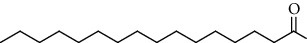
2	Erucic acid	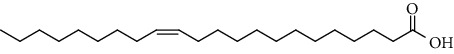
3	Linolenic acid	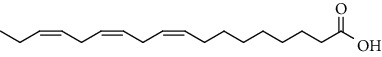
4	Pentadecanoic acid	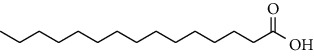

**Table 5:** Flavonoids isolated from genus *Urtica.*

Sr.	Name	
1.	2′, 4′, 5, 7, 8-Pentahy-droxyflavone; 7, 8-Di-Me ether	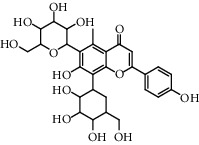
2.	Luteolin 7-*O*-neohes-peridoside	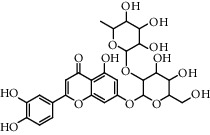
3.	Quercetin	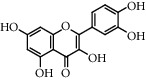
4.	Kaempferol	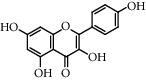
5.	Nicotiflorin	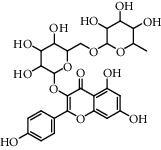
6.	Gossypetin	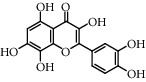
7.	Luteolin 7-O-b-d-Glucopyranoside	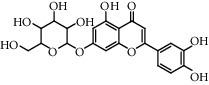
8.	Afzelin	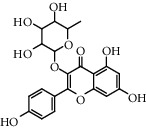
9.	Isovitexin	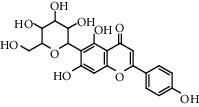
10.	Astragalin	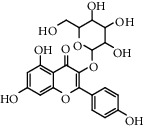

**Table 6:** Phenols extracted from *Urtica* spp.

Sr. no.	Compound name	Structural formula
1.	p-Coumaric acid	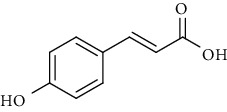
2.	Vanillic acid	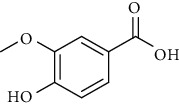
3.	4-Methoxybenzoic acid	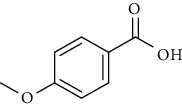
4.	Caffeoylmalic acid	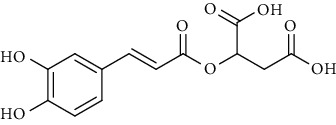
5.	Ferulic Acid	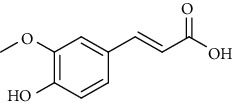
6.	Chlorogenic acid	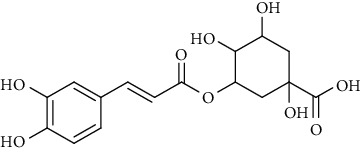
7.	Salicylic acid	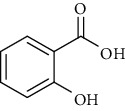
8.	Protocatechuic aldehyde	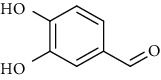
9.	Caffeic acid	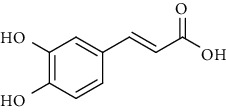

**Table 7:** Alcohols isolated from genus *Urtica*.

Sr.	Name	Chemical structure
1.	N-Tetracosanoylphytosphingosine	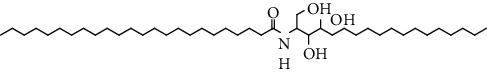
2.	Erythritol	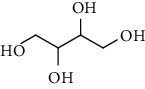
3.	1, 2, 3-Butanetriol	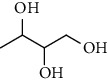
4.	14-Octacosanol	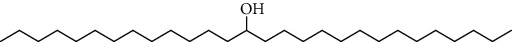

**Table 8:** Alkaloids isolated from genus *Urtica.*

Name	Chemical structure
Benzylisoquinoline	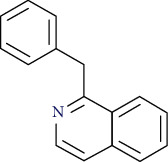
Chlorophyll A	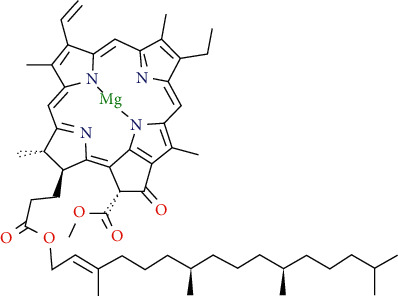
Chlorophyll B	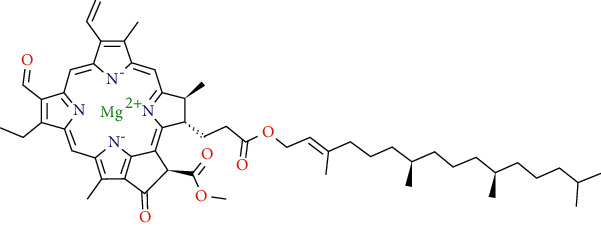

**Table 9:** Benzopyranoids isolated from genus *Urtica.*

Sr.	Name	Chemical structure
1.	6, 6′, 7, 7′-Tetrahy-droxy-[8, 8′-bi-2H-1-benzopyran]-2, 2′-dione; Tetra-Me ether	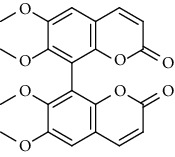
2.	7, 7′-dimethoxy-6, 6′-biscoumarin	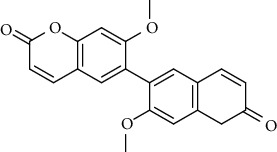
3.	Scopolin	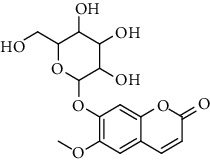
4.	6, 6′, 7, 7′-Tetrahydroxy-8,8′-bicoumarin; 6, 6′-Di-Me ether	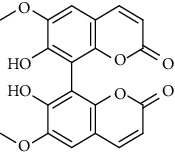
5.	Scoparone	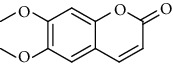
6.	Scopoletin	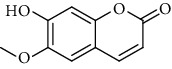

**Table 10:** Other compounds isolated from genus *Urtica.*

Sr.	Name	Chemical structure
1.	Tartaric acid	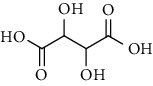
2.	Bis(5-formylfurfu-ryl) ether	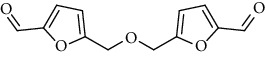
3.	Dotriacotane	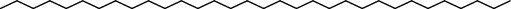
4.	2, 3-Dihydrobenzo-furan	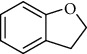
5.	Formic acid	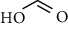
6.	Oxime- methoxy-phenyl	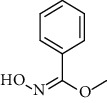
7.	1-Methoxy-4, 4a, 5, 6, 7, 8-hexahydro-2 (3H)-naphthalenone	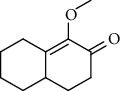
8.	Silane, triethyl (2-phenylethoxy)	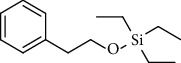
9.	*N, N*-Dimethyldo-decylamine	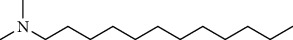
10.	Naphthalene	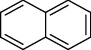

**Table 11:** Terpenoids isolated from genus *Urtica.*

Sr.	Name	Structural name
1.	3′-Hydroxyacetophenone	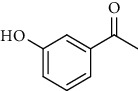
2.	4,7-Megastigma-diene-3, 9-diol; (3S,6R,7E,9R)-form, 3-Ketone, 9-*O*-[b-D-glucopyranosyl-(1 ⟶ 2)-b-d-glucopyranoside]	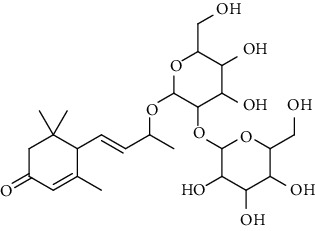
3.	1-(3, 4-Dihydroxyphe-nyl)-1, 2-propanediol; 3′-Me ether	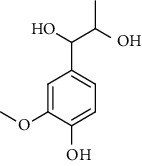
4.	(9Z,11E)-1, 3-hy-droxy-9, 11-octadeca-dienoic acid	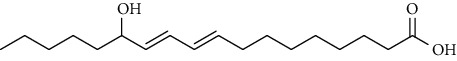
5.	Hexahydrofarnesyl acetone	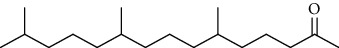
6.	Geranyl acetone	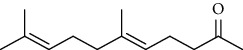
7.	(E)-Anethole	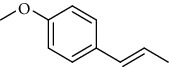
8.	*p*-Hydroxybenzalde-hyde	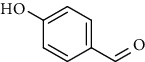
9.	b-Ionone	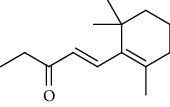

**Table 12:** Chemical composition of essential oil extracted of *Urtica pilulifera* and *Urtica diorca*.

NO	Compound	*Urtica pilulifera* (RT)	*Urtica diorca* (RI)	%
1	1-(4-Isopropylphenyl)-2-methylpropyl acetate	30.5829	—	2.062
2	1-(4′-pentenyl)-1, 2-epoxycyclopentane	24.2355	—	0.1271
3	1, 2-Benzenedicarboxylic acid	32.0424	—	13.5056
4	1, 4-Diazepine	27.0528	—	0.3009
5	1,8-Cineole	13.686	—	8.2085
6	1-Penten-3-one	27.5551	—	0.3782
7	2-(1-Pentenyl)furan	—	1056	0.29
8	2, 2, 6-Trimethylcyclohexanone	—	1035	0.28
9	2, 4, 6-Trimethyl-5*H*-1, 3, 5-dithiazine	—	1199	0.30
10	2-Methoxy-4-vinylphenol	12.3214	—	0.1087
11	2-Pentylfuran	—	991	0.84
12	2-Propenoic acid	5.302	—	2.2418
13	3, 5-Dimethyl-1, 2, 4-trithiolane	—	1134	0.30
14	3-Carene	15.8651	—	3.7624
15	3-Octanone	—	988	0.28
16	5, 6-Dihydro-4-pentyl-2, 6-dimethyl-4H-1, 3, 5-dithiazine	—	1588	0.57
17	5, 6-Dihydro-4-pentyl-2, 6-dimethyl-4H-1, 3, 5-dithiazine	—	1588	0.57
18	7-Acetyl-6-ethyl-1, 1, 4,4-tetramethyltetralin	32.5855	—	19.618
19	.alpha-Cetone	26.6658	—	0.9039
20	Anozol	29.1437	—	0.1346
21	Apoatropine	—	2093	0.82
22	Apoatropine	—	2093	0.82
23	Benzaldehyde	11.371	—	0.1391
24	Benzaldehyde	—	964	0.29
25	Benzofuranone	27.9014	—	0.1183
26	Benzoic acid	30.7118	—	0.873
27	Bicyclo[10.1.0]trideca-4, 8-diene-13-carboxamide	25.2741	—	0.1325
28	Bisabolene	—	1506	0.39
29	Bisomel	32.28	—	3.7872
30	Borneol	—	1171	0.31
31	Bornyl acetate	—	1283	2.14
32	Butyl acetate	6.7208	—	3.2399
33	Cadinene	—	1510	1.57
34	Cadinene	—	1516	2.37
35	Camphor	—	1145	0.27
36	Carvacrol	—	1299	0.30
37	Carvone	20.9362	—	0.1721
38	Citronellyl	19.7143	—	0.1388
39	Copaene-8-ol	—	1579	3.28
40	Decan-2-one	—	1192	0.28
41	Decanal	—	1206	0.29
42	Diethoxylated tridecyl alcohol	30.9494	—	0.1828
43	Ethylhexyl benzoate	31.187	—	0.3837
44	Farnesol	—	1715	1.88
45	Farnesol	—	1715	1.88
46	Farnesylacetone	—	1908	1.26
47	Farnesylacetone	—	1908	1.26
48	Furan-3-aldehyde	7.325	—	0.1458
49	Geranyl acetone	25.953	—	0.3483
50	Geranyl acetone	—	1448	2.22
51	Geranyl acetone	—	1448	2.22
52	Hexahydrofarnesylacetone	—	1844	31.20
53	Hexahydrofarnesylacetone	—	1844	31.20
54	Hexatriacontane	6.7208	—	11.5631
55	Humulene	—	1453	0.75
56	-Ionon *β*	26.822	—	0.1714
57	Ionone	—	1421	4.04
58	Ionone	—	1479	11.86
59	Isopropyl dodecanoate	—	1627	5.27
60	Isopropyl dodecanoate	—	1627	5.27
61	Lilyal	27.7113	—	1.8666
62	Limonene (−)-	13.5638	—	1.2463
63	Limonene (+)-	23.3733	—	6.7658
64	Menthol	—	1178	0.29
65	Methyl dihydrojasmonate	30.3181	—	0.8451
66	Methyl palmitate	—	1925	0.28
67	Methyl palmitate	—	1925	0.28
68	Methylbenzene	5.302	—	1.6415
69	Neophytadiene	32.3751	—	5.2683
70	n-Nonanal	15.9873	—	0.3288
71	n-Octanal	—	1004	0.30
72	Nonanal	—	1105	0.59
73	Ocimene	20.59	—	0.6869
74	Octanal	31.6555	—	2.0563
75	Octyl heptafluorobutyrate	17.9696	—	0.1347
76	p-Guaiacol	15.5392	—	0.1521
77	Phytol	—	2110	11.20
78	Phytol	—	2110	11.20
79	Safranal	—	1196	0.33
80	*β*-Selinene	—	1485	0.78
81	Terpinene	14.5413	—	0.1705
82	Thymol	—	1292	0.60
83	Trans-2,3-dimethylbicyclo[2.2.2]octane	22.4161	—	0.3454
84	Vanillin	21.812	—	1.7906
85	Vetivenene	—	1532	0.49
86	Vinyl	31.0445	—	0.3754
87	Xylene	8.3229	—	0.3848
88	*α*-Copaene-8-ol	—	1579	3.28
89	*α*-Humulene	—	1453	0.75
90	*α*-Ionone	—	1421	4.04
91	*α*-Longipinene	—	1347	0.30
92	*α*-Selinene	—	1493	0.70
93	*β*-2--Pinene	11.853	—	0.3957
94	*β*-Bisabolene	—	1506	0.39
95	*β*-Caryophyllene	—	1416	1.62
96	*β*-Cyclocitral	—	1217	0.35
97	*β*-Homocyclocitral	—	1254	0.28
98	*β*-Ionone	—	1479	11.86
99	*β*-Selinene	—	1485	0.78
100	*β*-Vetivenene	—	1532	0.49
101	*γ*-Cadinene	—	1510	1.57
102	*γ*-Terpinen	18.2615	—	0.3824
103	*γ*-Terpinene	18.6552	—	2.415
104	*δ*-Cadinene	—	1516	2.37

RI: retention time; RI: retention indices.

**Table 13:** *In vivo* studies of the genus *Urtica*.

Extract/compound	Doses	Route of administration	Model	Effect	Reference
Antiarthritis effect					
Total coumarins from *Urtica dentata* Hand	20, 40, 60 mg/kg	Orally every other day for 4 weeks after induction of arthritis	Collagen-induced arthritis BALB/c mice model	Dose-dependent ↓ arthritis score ↓paw swelling protect tissues against bone destruction ↓IFN-g, ↓IL-2 ↑IL-10, ↑TGF-B	[63]

Antioxidant effect					
Total 80% ethanolic extract of *Urtica dioica* L. leaves	50, 100 mg/kg	Orally daily for 14 days	Normal Swiss albino mouse model	↑cytochrome b5, ↑NADH-cytochrome b5 reductase, ↑glutathione S-transferase, ↑DT-diaphorase, ↑glutathione peroxidase, ↑glutathione reductase, ↑superoxide dismutase, ↑catalase ↓cytochrome P450, ↓lactate dehydrogenase, ↓NADPH-cytochrome P450 reductase, ↓total sulfhydryl groups, ↓nonprotein sulfhydryl groups, ↓protein-bound sulfhydryl groups	[64]

Antidiabetic effect					
Hexane, ethyl acetate and chloroform extracts of *Urtica pilulifera*	Two doses: 250 and 500 mg/kg	Orally daily for 4 weeks starting from day11 of diabetes induction	Streptozotocin and high-fat diet-induced type2 diabetes adult male albino rat model	Hypoglycemic effect - ethyl acetate and chloroform extracts ↓glucose level, ↓HbA1C, ↓ insulin resistance anti-inflammatory: ↓CRP, ↓TNF-*α* antioxidant: ↓MDA, ↑GSH, ↑SOD, ↑catalase	[65]
ZnO nanoparticles + aqueous extract of *Urtica dioica* leaves	ZnO + extract: 8 mg/dl	Intraperitoneally daily for 16 days	Alloxan-induced diabetic rat model	Both ZnO-extract and insulin (reference) ↓fasting blood glucose level in serum, while increased insulin level. ZnO-extract: ↑high-density lipoprotein ↓total cholesterol, ↓triglycerides	[66]

Antiendometriosis effect					
Hexane, ethyl acetate and methanol extracts of *Urtica dioica* L. aerial parts	100 mg/kg	Orally for 4 weeks	Surgery-induced endometriosis rat model	Methanol extract: ↓implant volumes, ↓adhesion scores ↓TNF-*α*, ↓VEGF, ↓IL-6; histopathological outcomes supported the results	[67]

Effect on prostate hyperplasia					
Polysaccharide fraction of *Urtica fissa*	62.5, 125, 250 mg/kg	Orally daily for 3 weeks	Testosterone propionate-induced prostate hyperplasia castrated rat model	↓prostate hyperplasia the lowest dose (62.5 mg/kg)-↓ indexes of wet weight,↓ dry weight , ↓volume by 17%, 23% and 32% highest dose (250 mg/kg)-↓indexes of wet weight, dry weight, ↓volume were further reduced by 25%, 33% and 37%; histopathological examination supported the results	[68]

Effect on nephrotoxicity					
Total 95% ethanol extract of *Urtica dioica*	Dose: 100 mg/kg	Orally daily for 10 days	Gentamicin-induced nephrotoxicity in male rabbit model	↓ serum creatinine, ↓blood urea, ↓ nitrogen antioxidant-↑glutathione, ↓malondialdehyde	[69]

Antiviral effect					
*Urtica dioica* agglutinin (UDA)	Three doses: 20, 10, 5 mg/kg	Intraperitoneally daily for 4 days	SARS-CoV- infected BALB/c mouse model	Treatment with UDA at dose 5 mg/kg significantly sheltered the mice against lethal infection with the virus but did not decrease virus titers in the lung; prevented weight loss and lung pathology scores of infected mice	[70]

Antiaging effect					
Total extract (50% ethanol) of *Urtica thunbergiana* leaves	Two doses: 0.1% and 1% g/kg of the animals' dry diet	Orally for 10 weeks	UVB-induced skin aging hairless mouse model	↓thinner and superficial wrinkles ↓erythema index ↑skin hydration; histopathological investigations supported the results	[71]

Anticancer effect					
Dichloromethane extract of *Urtica dioica*	Two doses: 10 and 20 mg/kg	Intraperitoneally daily for 28 days	4T1 (breast cancer cell line) allograft tumor BALB/c mouse model	↓tumor size and weight. ↑apoptosis, ↓proliferation. ↓Bcl2, ↑caspase 3; histopathology examinations supported the results	[72]

Symbols: ↑ increase, ↓decrease.

## Data Availability

The data supporting this review were taken from previously reported studies and datasets, which have been cited. The processed data are available from the corresponding author upon request.
